# PD-1/PD-L1抑制剂在非小细胞肺癌中的临床研究进展

**DOI:** 10.3779/j.issn.1009-3419.2019.07.06

**Published:** 2019-07-20

**Authors:** 思璇 吴, 春宏 胡, 芳 吴, 元强 吴, 平 刘

**Affiliations:** 410011 长沙，中南大学湘雅二医院肿瘤科 Department of Oncology, the Second Xiangya Hospital, Central South University, Changsha 410011, China

**Keywords:** 肺肿瘤, 肿瘤免疫治疗, PD-1/PD-L1, 免疫检查点抑制剂, Lung neoplasms, Cancer immunotherapy, PD-1/PD-L1, Immune checkpoint inhibitor

## Abstract

非小细胞肺癌（non-small cell lung cancer, NSCLC）是肺癌最常见的病理类型。近年来，免疫治疗迅速发展，免疫检查点抑制剂尤其是程序性死亡因子-1（programmed death-1, PD-1）/程序性死亡因子配体-1（programmed death-ligand 1, PD-L1）抑制剂已在NSCLC的治疗中取得突破性进展，改变了NSCLC治疗的格局。以PD-1/PD-L1为靶点的免疫检查点抑制剂无论在晚期NSCLC的一线和二线治疗，局部晚期NSCLC的辅助治疗，还是早期NSCLC的新辅助治疗中均为患者带来获益，在NSCLC的综合治疗中显示出重要地位。本文针对以PD-1/PD-L1为靶点的免疫检查点抑制剂在NSCLC中的临床研究进展展开综述。

## Abstract (en)

Non-small cell lung cancer (NSCLC) is the most common pathological type of lung cancer. In recent years, immunotherapy has developed rapidly. Immune checkpoint inhibitors, especially programmed death-1 (PD-1)/programmed death-ligand 1 (PD-L1) inhibitors have made a breakthrough in the treatment of NSCLC and changed the treatment patern for NSCLC. Immunological checkpoint inhibitors targeting PD-1/PD-L1 are benefcial to patients, both in the frst-line and second-line treatment of advanced NSCLC, in the adjuvant treatment of locally-advanced NSCLC, and in the neoadjuvant therapy of early NSCLC, which show an important role in the comprehensive treatment of NSCLC. Tis article reviews the clinical research progress of immunological checkpoint inhibitors targeting PD-1/PD-L1 in NSCLC.

肺癌是全球范围内发病率及致死率最高的恶性肿瘤^[1]^，其中，非小细胞肺癌（non-small cell lung cancer, NSCLC）占80%-85%。NSCLC患者预后差，Ⅰ期患者5年生存率为68%-92%，Ⅱ期为53%-60%，Ⅲ期为13%-36%，Ⅳ期仅为0%-10%^[2]^，亟待寻求更有效的治疗方法。免疫治疗包括肿瘤疫苗、单克隆抗体、肿瘤过继免疫治疗及免疫增强剂等。在NSCLC的免疫治疗中研究最多的为程序性死亡因子-1（programmed death-1, PD-1）/程序性死亡因子配体-1（programmed death-ligand 1, PD-L1）抑制剂。PD-1是延伸的CD28/CTLA-4家族T细胞调节因子和蛋白质的成员，主要表达在成熟T细胞上^[3]^，其配体包括PD-L1和PD-L2，PD-L1主要表达在肿瘤细胞上^[4]^。PD-1和PD-L1的结合会抑制CD4^+^ T细胞和CD8^+^ T细胞的增殖和活性，使其减少对周围组织的免疫应答并防止自身免疫疾病的发生^[5]^，但在肿瘤中，它减少肿瘤局部微环境中T细胞的自身免疫杀伤功能，导致肿瘤免疫逃逸和促进肿瘤生长^[6]^。因此，抑制PD-1和PD-L1的结合可增强T细胞的增殖和杀伤功能。已有大量临床研究证实了PD-1/PD-L1抑制剂在NSCLC治疗中的有效性（表 1），本文针对PD-1/PD-L1抑制剂在NSCLC中的研究进展展开综述。

**1:** PD-1/PD-L1抑制剂在NSCLC一线、二线及辅助治疗中的临床研究 Clinical researches of PD-1/PD-L1 inhibitors in first-line, second-line and adjuvant therapy with NSCLC

Regimen	Trials	Types of NSCLC	PD-L1 selection	Line of therapy	ORR	mPFS (mon)	mOS (mon)
Nivolumab	CheckMate-017	Squamous	None	2^nd^	20.00%	3.5 (HR=0.62)	9.2 (HR=0.59)
Nivolumab	CheckMate-057	Non-squamous	None	≥2^nd^	19.00%	2.3 (HR=0.92)	12.2 (HR=0.73)
Nivolumab	CheckMate-078	All	None	2^nd^	16.90%	2.8 (HR=0.77)	11.9 (HR=0.64)
Nivolumab	CheckMate-026	All	≥5%	1^st^	26.00%	4.2 (HR=1.15)	14.4 (HR=1.02)
Nivolumab+ Ipilimumab	CheckMate-227	All	None	1^st^	45.30%	7.2 (HR=0.58)	/
Pembrolizumab	Keynete-010	All	≥1% (2 mg/kg)	≥2^nd^	18.00%	3.9 (HR=0.88)	10.4 (HR=0.71)
			≥1% (10 mg/kg)		18.00%	4.0 (HR=0.79)	12.7 (HR=0.61)
			≥50% (2 mg/kg)		29.00%	5.0 (HR=0.59)	14.9 (HR=0.54)
			≥50% (10 mg/kg)		30.00%	5.2 (HR=0.59)	17.3 (HR=0.50)
Pembrolizumab	Keynote-024	All	≥50%	1^st^	44.80%	10.3 (HR=0.50)	30.0 (HR=0.63)
Pembrolizumab	Keynote-042	All	≥1%	1^st^	27.30%	5.4 (HR=1.07)	16.7 (HR=0.81)
			≥20%		33.40%	6.2 (HR=0.94)	17.7 (HR=0.77)
			≥50%		39.50%	7.1 (HR=0.81)	20.0 (HR=0.69)
Pembrolizumab	Keynote-021	Non-squamous	All population	1^st^	56.70%	24.0 (HR=0.53)	NR (HR=0.56)
			< 1%		57.00%	/	/
			≥1%		54.00%		
			1%-49%		26.00%		
			≥50%		80.00%		
Pembrolizumab	Keynote-189	Non-squamous	All population	1^st^	47.60%	8.8 (HR=0.52)	NR (HR=0.49)
			< 1%		32.30%	/	HR=0.59
			1%-49%		48.40%		HR=0.55
			≥50%		61.40%		HR=0.42
Pembrolizumab	Keynote-407	Squamous	All population	1^st^	57.90%	6.4 (HR=0.56)	15.9 (HR=0.64)
			< 1%		63.20%	6.3 (HR=0.68)	HR=0.61
			≥1%		/	/	HR=0.65
			1%-49%		49.50%	7.2 (HR=0.56)	HR=0.57
			≥50%		60.30%	8.0 (HR=0.37)	HR=0.64
Atezolizumab	POPLAR	All	None	2^nd^	17.00%	2.7 (HR=0.94)	12.6 (HR=0.73)
Atezolizumab	OAK	All	None	≥2^nd^	14.00%	2.7 (HR=0.96)	13.8 (HR=0.73)
Atezolizumab	IMpower130	Non-squamous	All population	1^st^	49.20%	7.0 (HR=0.64)	18.6 (HR=0.79)
			< 1%		/	6.2 (HR=0.72)	15.2 (HR=0.81)
			1%-49%			8.3 (HR=0.61)	23.7 (HR=0.70)
			≥50%			6.4 (HR=0.51)	17.3 (HR=0.84)
Atezolizumab	IMpower131	Squamous	All population	1^st^	49.00%	6.3 (HR=0.71)	14.0 (HR=0.96)
			< 1%		44.00%	5.7 (HR=0.81)	/
			1%-49%		52.00%	6.0 (HR=0.70)	
			≥50%		60.00%	10.1 (HR=0.44)	
Atezolizumab	IMpower132	Non-squamous	All population	1^st^	47.00%	7.6 (HR=0.60)	18.1 (HR=0.81)
			< 1%		44.00%	8.5 (HR=0.45)	/
			1%-49%		38.00%	6.2 (HR=0.80)	
			≥50%		72.00%	10.8 (HR=0.46)	
Atezolizumab	IMpower150	Non-squamous	All population	1^st^	56.00%	8.3 (HR=0.62)	19.2 (HR=0.78)
			< 1%		/	7.1 (HR=0.77)	25.2 (HR=0.70)
			1%-49%			8.3 (HR=0.56)	20.3 (HR=0.80)
			≥50%			12.6 (HR=0.39)	17.1 (HR=0.82)
Avelumab	JAVELIN Lung 200	All	≥1%	2^nd^	19.00%	3.4 (HR=1.01)	11.4 (HR=0.90)
Durvalumab	PACIFIC	All	None	Adjuvant therapy	28.40%	16.8 (HR=0.52)	NR (HR=0.68)
NSCLC: non-small cell lung cancer; PD-1: programmed death-1; PD-L1: programmed death-ligand 1; mPFS: mean progression-free survival; mOS: mean overall survival; NR: not reach.							

## 1 PD-1/PD-L1抑制剂用于晚期NSCLC的二线治疗

### 1.1 Nivolumab

CheckMate-017^[7]^Ⅲ期临床研究入组了272例以铂类为基础的化疗后进展的肺鳞癌患者，nivolumab组和多西他赛组的中位无进展生存期（progression-free survival, PFS）分别为3.5个月和2.8个月（HR=0.62, *P* < 0.001），中位总生存期（overall survival, OS）分别为9.2个月和6.0个月（HR=0.59, *P* < 0.001），客观缓解率（objective response rate, ORR）分别为20%和9%（*P*=0.008）。CheckMate-057^[8]^Ⅲ期临床研究入组了582例以铂类为基础的化疗后进展的非鳞癌患者，nivolumab组和多西他赛组的中位OS分别为12.2个月和9.4个月（HR=0.73, *P*=0.002），ORR分别为19%和12%（*P*=0.02），而两组中位PFS无显著差异（2.3个月*vs* 4.2个月）（HR=0.92, *P*=0.39）。两项研究长期随访结果显示，免疫治疗组患者的3年OS率为17%^[9]^。更早进行的Ⅰ期CA209-003研究显示，nivolumab单药用于≥二线NSCLC患者的5年OS率为16%，其中鳞癌（16%）与非鳞癌（15%）患者的生存率相似^[10]^，以上均显示了免疫治疗的“拖尾效应”（在部分患者中持续长久的疗效）。

CheckMate-078研究是一项以中国患者为主的Ⅲ期临床研究，共入组了451例（占总人数的89.5%）EGFR/ALK野生型含铂双药化疗后进展的中国NSCLC患者，nivolumab组和多西他赛组的ORR分别为16.9%与3.4%（*P* < 0.000, 1），与多西他赛组相比，nivolumab组显著延长了患者的中位OS（11.9个月*vs* 9.0个月）（HR=0.64, *P*=0.000, 4），不同PD-L1表达水平及不同组织学类型均可见OS获益。

以上研究中，nivolumab对比多西他赛均有OS获益，延长患者OS约3个月，nivolumab组ORR约为17%-20%，但PFS获益不一。CheckMate-017/078研究显示患者预后与PD-L1的表达无关，而CheckMate-057研究提示PD-L1表达可预测患者预后。基于CheckMate-017与CheckMate-057研究，美国食品药品监督管理局（Food and Drug Administration, FDA）分别于2015年3月4日和2015年10月9日批准了nivolumab用于治疗经铂为基础化疗后疾病进展的转移性鳞状与非鳞状NSCLC。基于CheckMate-078研究，我国国家药品监督管理局于2018年6月15日批准了nivolumab用于治疗EGFR/ALK野生型的含铂化疗后进展的NSCLC。Nivolumab成为第一个改写了NSCLC二线治疗格局的免疫治疗药物。

### 1.2 Pembrolizumab

Keynote-010^[11]^Ⅲ期临床研究入组了1, 034例一线治疗进展后的PD-L1≥1%的晚期NSCLC患者。Pembrolizumab 2 mg/kg组、pembrolizumab 10 mg/kg组及多西他赛组的中位OS分别为10.4个月（HR=0.71, *P*=0.000, 8）、12.7个月（HR=0.61, *P* < 0.000, 1）与8.5个月。研究显示PD-L1表达可预测pembrolizumab的疗效，在PD-L1≥50%的患者中，这三组患者的中位OS分别为14.9个月（HR=0.54, *P*=0.000, 2）、17.3个月（HR=0.50, *P* < 0.000, 1）和8.2个月。2018年世界肺癌大会（World Conference on Lung Cancer, WCLC）上公布了在我国进行的Ⅰ期Keynote-032研究的结果，该研究入组了42例中国晚期NSCLC二线治疗以后的患者，患者随机接受不同剂量组的pembrolizumab治疗，总体人群ORR为14.3%，中位PFS为2.3个月，与国外的研究数据相似，PK数据也与国外研究的数据一致，主要不良反应仍然是与免疫相关的副反应，初步显示了pembrolizumab二线治疗NSCLC在中国患者中的安全性及有效性，且与西方人群数据一致。

基于Keynote-010研究，2016年10月24日美国FDA批准pembrolizumab用于治疗在以铂类药物为基础的化疗后进展的PD-L1≥1%的转移性NSCLC。在我国进行的Ⅰ期Keynote-032研究初步显示了pembrolizumab二线治疗NSCLC在中国患者的安全性及有效性，评估pembrolizumab二线治疗中国晚期NSCLC患者疗效的Ⅲ期临床试验Keynote-033也在进行之中。

### 1.3 Atezolizumab

POPLAR^[12]^Ⅱ期临床研究入组了287例晚期NSCLC患者，对比了atezolizumab与多西他赛二、三线治疗的疗效和安全性。结果显示，atezolizumab组和多西他赛组的中位OS分别为12.6个月和9.7个月（HR=0.73, *P*=0.04），中位PFS无显著差异（2.7个月*vs* 3.0个月；HR=0.94）。2018年欧洲肺癌大会（European Lung Cancer Congress, ELCC）报道了POPLAR试验3年OS结果，atezolizumab组3年OS率较多西他赛组明显增高（18.7% *vs* 10.0%）。Ⅲ期临床研究OAK^[13]^入组了1, 225例一线治疗失败的Ⅲb期/Ⅳ期NSCLC患者，结果显示：atezolizumab组和多西他赛组的中位OS分别为13.8个月和9.6个月（HR=0.73, *P*=0.000, 3），中位PFS无获益（2.7个月*vs* 3.8个月；HR=0.96）。基于以上研究结果，2016年10月18日美国FDA批准了atezolizumab用于治疗以铂类药物为基础的化疗后进展的晚期NSCLC。

### 1.4 Avelumab

2018年WCLC公布了Ⅲ期JAVELIN Lung 200临床研究的结果^[14]^。该研究入组了792例含铂化疗后进展的晚期NSCLC，分别使用avelumab与多西他赛二线治疗，在PD-L1≥1%的患者中，avelumab组与多西他赛组的中位OS分别为11.4个月与10.3个月（HR=0.90, *P*=0.16），未达到主要研究终点。

PD-1/PD-L1抑制剂已成为晚期NSCLC二线治疗的标准治疗。在各项研究中，患者PFS获益不一致，OS基本均有获益，其中少部分患者（约20%）可持续获益，无论组织学类型均有获益，PD-L1表达对免疫治疗疗效预测有一定的指导意义。其中多项研究的亚组分析显示，免疫治疗并不能给驱动基因阳性患者带来更大的获益。

## 2 PD-1/PD-L1抑制剂用于晚期NSCLC的一线治疗

### 2.1 单药免疫治疗

CheckMate-026^[15]^Ⅲ期临床研究入组423例PD-L1≥5%的初治NSCLC患者，对比nivolumab与多西他赛治疗的疗效，nivolumab组与多西他赛组的中位PFS无显著差异（4.2个月*vs* 5.9个月）（HR=1.15; 95%CI: 0.91-1.45），中位OS也无显著差异（14.4个月*vs* 13.2个月）（HR=1.02; 95%CI: 0.80-1.30），ORR分别为26%与33%。研究进一步分析发现高肿瘤突变负荷（tumor mutational burden, TMB）亚组患者ORR较化疗组明显升高（47% *vs* 28%），且中位PFS明显延长（9.7个月*vs* 5.8个月）（HR=0.62, 95%CI: 0.38-1.00），但中位OS无获益。

Keynote-024^[16]^Ⅲ期临床研究纳入了305例晚期初治PD-L1≥50%的EGFR/ALK野生型的NSCLC患者，对比pembrolizumab与化疗治疗的疗效。结果显示，两组的ORR分别为44.8% *vs* 27.8%，对比化疗，pembrolizumab组的中位PFS延长4.3个月（10.3个月*vs* 6.0个月，HR=0.50，*P* < 0.001），中位OS延长15.8个月（30.0个月*vs* 14.2个月，HR=0.63，*P*=0.002）。基于该研究，2016年10月24日美国FDA批准pembrolizumab用于PD-L1≥50%、EGFR及ALK野生型的转移性NSCLC患者的一线治疗。这项“地震”式的研究彻底改写了晚期NSCLC一线治疗的格局。

Pembrolizumab可单药应用于PD-L1≥50%的晚期NSCLC患者，那么是否能扩大用于更广泛的人群？Ⅲ期Keynote-042研究给出了答案^[17]^。该研究纳入了1, 274例晚期初治且无*EGFR*/*ALK*突变的PD-L1≥1%的NSCLC患者，结果显示无论在PD-L1≥1%、≥20%或≥50%的患者中，pembrolizumab较化疗均有OS获益。但需要注意的是该研究中，PD-L1≥50%的患者约占总人群的50%（远高于其他研究），亚组分析的结果提示PD-L1表达在1%-49%之间的患者中，pembrolizumab较化疗并没有明显OS获益，所以，真正获益的人群还是PD-L1≥50%的患者。因此该研究最终并未改写指南。

在单药一线免疫治疗中，呈现出结果不一的局面。Nivolumab在PD-L1≥5%的NSCLC患者中，OS较标准化疗无获益，而pembrolizumab在PD-L1≥50%的患者中，OS明显获益，提示一线免疫单药治疗需高度选择患者。虽然CheckMate-026研究未达到主要研究终点，但进一步回顾性分析显示TMB与患者疗效密切相关。TMB对nivolumab与PD-L1对pembrolizumab的ORR及PFS的预测作用相似，但TMB不能很好地预测OS，所以，PD-L1作为疗效预测分子标志物更为确切。

### 2.2 免疫联合治疗

#### 2.2.1 联合化疗

Keynote-021^[18]^Ⅱ期临床研究纳入了123例晚期非鳞癌患者，比较pembrolizumab联合培美曲塞和卡铂与单纯化疗一线治疗晚期NSCLC的疗效和安全性。结果显示：联合组与化疗组的ORR分别为56.7%和30.2%（*P*=0.001, 6），中位PFS分别为24.0个月与9.3个月（HR=0.53, *P*=0.004, 9），联合组的中位OS未达到（NR *vs* 21.1个月，HR=0.56，*P*=0.015, 1）。Keynote-189^[19]^Ⅲ期临床研究入组了616例晚期非鳞癌患者，pembrolizumab联合培美曲塞和卡铂/顺铂与单纯化疗组的ORR分别为47.6%与18.9%，中位PFS分别为8.8个月与4.9个月（HR=0.52, *P* < 0.000, 01），联合组的中位OS未达到（NR *vs* 11.3个月，HR=0.49，*P* < 0.001），卡铂和顺铂组患者预后没有显著差异。Keynote-407^[20]^Ⅲ期临床研究纳入了559例初治晚期鳞癌患者，评估pembrolizumab联合紫杉醇/白蛋白紫杉醇和卡铂一线治疗的疗效，结果显示：联合组显著提高了患者的ORR（57.9% *vs* 38.4%），延长了患者的中位OS（15.9个月*vs* 11.3个月）（HR=0.64, *P* < 0.001），同时也改善了患者的中位PFS（6.4个月*vs* 4.8个月）（HR=0.56, *P* < 0.001）。

IMpower130是一项Ⅲ期临床研究，该研究纳入了723例初治晚期非鳞癌患者，评估atezolizumab联合白蛋白紫杉醇和卡铂治疗与单纯化疗治疗的疗效，联合组与单纯化疗组的中位PFS分别为7.0个月与5.5个月（HR=0.64, *P* < 0.000, 1），中位OS分别为18.6个月与13.9个月（HR=0.79, *P* < 0.033），ORR分别为49.2%与31.9%，达到了主要终点PFS与OS。IMpower132是一项评估atezolizumab联合培美曲塞和卡铂/顺铂治疗与单纯化疗治疗晚期非鳞癌疗效的Ⅲ期临床研究，联合组与单纯化疗组的PFS分别为7.6个月和5.2个月（HR=0.60, *P* < 0.000, 1），OS分别为18.1个月与13.6个月（HR=0.81, *P*=0.079, 7），ORR分别为47%与32%，主要终点PFS达到，OS未达到。IMpower131是一项评估atezolizumab联合紫杉醇/白蛋白紫杉醇和卡铂治疗与单纯化疗治疗晚期肺鳞癌疗效的Ⅲ期临床研究，联合组与单纯化疗组的中位PFS分别为6.3个月和5.6个月（HR=0.71, *P*=0.000, 1），没有观察到OS显著差异（HR=0.96, *P*=0.693, 1）。

Pembrolizumab联合化疗一线治疗晚期NSCLC，均有PFS及OS获益，pembrolizumab组ORR约为48%-58%，无论PD-L1水平如何，患者均可从联合治疗中获益。而atezolizumab联合化疗组PFS及OS获益不一，atezolizumab组ORR约为47%-49%。在atezolizumab联合化疗治疗非鳞癌患者的研究中，Impower130研究使用atezolizumab联合白蛋白紫杉醇和卡铂治疗，Impower132研究使用atezolizumab联合培美曲塞和卡铂/顺铂治疗，Impower130研究有OS获益，约延长患者5个月的OS，Impower132研究OS无临床意义，提示不同的免疫治疗药物联合不同的化疗药物疗效可能不同，需要进一步探索找寻最佳联合治疗方式。基于Keynote-021研究，美国FDA于2017年5月10日批准了pembrolizumab联合培美曲塞和卡铂一线治疗*EGFR*/*ALK*基因野生型的转移性非鳞状NSCLC。基于Keynote-189研究，美国FDA于2018年8月20日批准了pembrolizumab联合培美曲塞和铂类作为*EGFR*/*ALK*基因野生型的转移性非鳞状NSCLC患者的一线治疗。基于Keynote-407研究，美国FDA于2018年10月30日了批准pembrolizumab联合卡铂和紫杉醇/白蛋白紫杉醇作为转移性鳞状NSCLC的一线治疗药物。

#### 2.2.2 联合抗血管生成药物

IMpower150^[21]^Ⅲ期临床研究入组了1, 202例初治的晚期非鳞癌患者，评估atezolizumab+化疗（紫杉醇与卡铂）联合或不联合贝伐珠单抗治疗的疗效和安全性，atezolizumab+化疗+贝伐珠单抗组与化疗+贝伐珠单抗组的中位PFS分别为8.3个月与6.8个月（HR=0.62, *P* < 0.001），中位OS分别为19.2个月与14.7个月（HR=0.78, *P*=0.02），ORR分别为56%与41%。对于不同PD-L1表达状态的非鳞癌患者，atezolizumab+化疗+贝伐珠单抗组的PFS和OS均获益。2018年12月6日，美国FDA批准atezolizumab+紫杉醇与卡铂+贝伐珠单抗联合用于*EGFR*/*ALK*基因野生型的转移性非鳞状NSCLC患者的一线治疗。

#### 2.2.3 联合免疫治疗

CheckMate-227^[22]^Ⅲ期临床研究入组了299例晚期NSCLC患者，共分为4组：含铂双药化疗组、nivolumab组、nivolumab+ipilimumab组、nivolumab+含铂双药化疗组。结果显示，nivolumab+ipilimumab组与单纯化疗组的中位PFS分别为7.2个月与5.5个月（HR=0.58, *P* < 0.001）。TMB≥10 mut/Mb的患者的ORR、PFS、OS均优于化疗组，而PFS与PD-L1表达无关。结果表明，nivolumab+ipilimumab可以作为高TMB晚期NSCLC患者的一线治疗新选择，TMB可能作为nivolumab+ipilimumab治疗的疗效预测标志物。

IMpower150研究首次在*EGFR*/*ALK*突变阳性的亚组患者中观察到PFS获益（9.7个月*vs* 6.1个月，HR=0.59，95%CI: 0.37-0.94）。Keynote-189研究中OS的HR值为0.49，而IMpower130及IMpower150研究中OS的HR值分别为0.79与0.78，提示不同的免疫治疗药物之间存在疗效差异。其中，pembrolizumab显示出更好的临床获益。因此，美国国立综合癌症网络（National Comprehensive Cancer Network, NCCN）/欧洲肿瘤内科学会（European Society for Medical Oncology, ESMO）/肿瘤免疫治疗学会（Society for Immunotherapy of Cancer, SITC）指南均推荐pembrolizumab联合化疗作为一线治疗非鳞状EGFR/ALK野生型NSCLC的首选。对于PD-L1≥50%的晚期NSCLC，NCCN指南推荐优先选择单药免疫治疗，也可选择免疫联合化疗的策略。Keynote-024、Keynote-042及Keynote-189研究显示，在PD-L1≥50%的NSCLC患者中，免疫联合治疗较单药治疗带来更高的肿瘤缓解率，更低的死亡风险（HR分别为0.63、0.69与0.42），但毒副反应更大，目前仍缺乏头对头的前瞻性研究比较两者的生存获益，在PD-L1 < 50%的人群中，联合治疗是更合适的治疗策略。

## 3 PD-1/PD-L1抑制剂用于局部晚期NSCLC的辅助治疗

放疗可以通过多种途径影响免疫系统，包括释放新抗原、上调肿瘤细胞PD-L1表达、招募T细胞至肿瘤灶、抵消肿瘤微环境中的免疫抑制信号。临床前研究显示PD-1/PD-L1抑制剂与放疗具有协同作用。

PACIFIC^[23]^Ⅲ期临床研究入组了713例Ⅲ期NSCLC患者，在同步放化疗的基础上，对患者进行PD-L1抑制剂durvalumab的辅助治疗，与对照组相比，中位PFS延长了11.2个月（16.8个月*vs* 5.6个月）（HR=0.52, *P* < 0.001）。在2018年的WCLC上，PACIFIC研究更新了OS数据，研究结果显示，对照组中位OS为28.7个月，研究组中位OS尚未达到（HR=0.68, *P*=0.002, 51），即durvalumab辅助治疗可以明显延长患者OS，亚组分析中显示，PD-L1 < 1%的患者中，durvalumab辅助治疗组与同步放化疗组并无明显差异。同步放化疗一直是局部晚期NSCLC患者的标准治疗，“海啸”般的PACIFIC研究彻底改写了这类患者的治疗格局。2018年2月16日，美国FDA批准了durvalumab用于无法切除的Ⅲ期NSCLC患者的辅助治疗，这些患者在接受同步放化疗后疾病未进展。

2018年美国临床肿瘤学会（American Society of Clinical Oncology, ASCO）报道了一项Ⅱ期临床研究LUN14-179的结果，该研究入组了93例同步放化疗后无进展的Ⅲ期NSCLC患者，予以pembrolizumab辅助治疗1年。结果显示：中位疾病转移或死亡时间为30.7个月，中位PFS为15.0个月，中位OS尚未达到，进一步证明了局部晚期NSCLC患者同步放化疗联合辅助免疫治疗的安全性与有效性。

## 4 PD-1/PD-L1抑制剂用于早期NSCLC的新辅助治疗

乳腺癌动物模型中，新辅助免疫治疗较辅助免疫治疗具有更高的总体存活率^[24]^，其原因为疾病早期可能拥有更完整的免疫系统功能，将检查点抑制剂用于早期疾病的治疗中可能达到更好的疗效^[25]^。同时，新辅助免疫治疗在其他肿瘤（如恶性黑色素瘤）中已被证实有较好的临床获益^[26]^。在肺癌中，化疗新辅助治疗疗效有限，单纯化疗新辅助治疗出现病理完全缓解（pathological complete response, pCR）非常少见，而一些临床研究的结果提示免疫新辅助治疗疗效较佳（表 2）。下面我们将对肺癌新辅助免疫治疗展开阐述。

**2:** PD-1/PD-L1抑制剂在NSCLC新辅助治疗中的临床研究（已有五项研究结果汇总） Clinical researches of PD-1/PD-L1 inhibitors in neoadjuvant therapy with NSCLC (summary of five research results)

Regimen	Trials	Patients phase	Phase	Population	Radiographic responses	MPR	pCR	AE
Nivolumab	CheckMate-159	Ⅰ-Ⅲa	Ⅱ	21	10% (2/21)	45% (9/20)	15% (3/20)	Grade3/4: 1
Atezolizumab	LCMC3	Ib-Ⅲb	Ⅱ	54	7% (3/45)	22% (10/45)	7% (3/45)	Grade3/4: 3
Atezolizumab+nab-paclitaxe and carboplatin	NCT02716038	Ib-Ⅲa	Ⅱ	18	57% (8/14)	50% (7/14)	21% (3/14)	Grade3/4: 12
Nivolumab+carboplatin and paclitaxel	NADIM	Ⅲa	Ⅱ	46	70% (21/30)	80% (24/30)	60% (18/30)	Grade3/4: 6
Nivolumab±Ipilimumab	NEOSTAR	Ⅰ-Ⅲa	Ⅱ	36	22% (7/32)	31% (8/26)	19% (5/26)	Grade3/5: 3
MPR: major pathological response; pCR: pathological complete response; AE: adverse event.								

### 4.1 单药新辅助免疫治疗

CheckMate-159^[27]^Ⅱ期临床研究入组了21例可手术的早期（Ⅰ期-Ⅲa期）NSCLC患者，术前接受2个周期的nivolumab治疗，主要终点为安全性与可行性。结果显示: 20例患者完成手术，新辅助治疗安全性良好，并不延迟手术进行。Nivolumab新辅助免疫治疗后2例（10%）患者影像学缓解，3例（15%）患者达到pCR（但其中1例患者肺门淋巴结残留癌细胞），9例（45%）患者达到了重大病理缓解（major pathological response, MPR）（手术切除的肿瘤原发灶中≤10%活细胞）。同时也发现了病理学缓解与PD-L1表达无关，与治疗前TMB水平显著相关。LCMC3是一项Ⅱ期临床研究，入组了54例可手术的早期（Ib期-Ⅲb期）NSCLC患者，术前行两周前atezolizumab新辅助免疫治疗，术后行12个月atezolizumab辅助治疗，结果显示：45例患者完成手术，术前影像学评估3例（7%）患者缓解，术后评估3例（7%）患者达到了pCR，10例（22%）患者达到了MPR，PD-L1阴性亚组无pCR或MPR。以上两项试验中MPR缓解率均显著高于术前影像学缓解率，说明目前影像学在评估免疫新辅助治疗疗效上存在不足。目前还有很多类似研究正在进行（表 3），如评估atezolizumab新辅助治疗NSCLC患者疗效的Ⅱ期临床试验NCT02994576，评估pembrolizumab新辅助治疗NSCLC疗效的Ⅱ期临床研究NCT03197467及评估durvaluma新辅助治疗NSCLC的Ⅱ期临床研究NCT03030131等。

**3:** PD-1/PD-L1抑制剂在NSCLC新辅助治中的临床研究（正在进行） Ongoing clinical researches of PD-1/PD-L1 inhibitors in neoadjuvant therapy with NSCLC

NCT number	Phase	Arms	Key endpoints	Population
Pembrolizumab				
NCT02987998	Ⅰ	Neoadjuvant pembrolizumab with chemoradiotherapy and adjuvant pembrolizumab	Safety, ORR, PFS, ORR, pCR, OS	20
NCT02938624	Ⅰ	Neoadjuvant pembrolizumab	Dose limiting toxicities	28
NCT03217071	Ⅱ	2 cycles of neoadjuvant pembrolizumab with SBRT	Change in number of infiltrating CD3^+^ T cells, OS	40
NCT02818920	Ⅱ	Two doses of neoadjuvant pembrolizumab and four doses of adjuvant nivolumab	Surgical feasibility, ORR, DFS, AE	32
NCT03197467	Ⅱ	2 cycles of neoadjuvant pembrolizumab	AE	30
NCT03425643	Ⅲ	4 cycles of neoadjuvant pembrolizumab and chemotherapy+adjuvant pembrolizumab	EFS, OS	786
Atezolizumab				
NCT02994576	Ⅱ	Neoadjuvant atezolizumab	MPR	60
NCT03456063	Ⅲ	4 cycles of neoadjuvant atezolizumab+adjuvant atezolizumab	MPR, EFS, OR, pCR, MPR	302
Durvalumab				
NCT03030131	Ⅱ	3 cycles of neoadjuvant durvalumab	Surgical resection R0, response rate, MPR	81
NCT02572843	Ⅱ	3 cycles of neoadjuvant cisplatin and docetaxel, followed by 2 cycles of durvalumab and adjuvant durvalumab	EFS, OS, OR, pCR, AE	68
Nivolumab+Ipilimumab				
NCT02259621	Ⅱ	Neoadjuvant nivolumab±ipilimumab	Safety, feasibility, pathologic reponse, radiographic response	30
NCT02998528	Ⅰ-	Neoadjuvant nivolumab+ ipilimumab	EFS, pCR, OS, MPR	350
Durvalumab+Tremelimumab				
NCT03237377	Ⅱ	2 cycles of neoadjuvant durvalumab+tremelimumab with radiotherapy	Safety, surgical mortality	32
DFS: disease free survival; EFS: Event-free survival.				

### 4.2 联合新辅助免疫治疗

NADIM为一项Ⅱ期临床研究，该研究入组了46例可手术的Ⅲa期NSCLC患者，术前行3个周期nivolumab联合紫杉醇和卡铂新辅助治疗，术后行12个月的nivolumab辅助治疗。2018年WCLC上报道了30例患者的数据，术前21例（70%）患者达到了影像学缓解，术后18例（60%）患者达到了pCR，24例（80%）患者达到了MPR，治疗安全性良好，并不延迟手术进行。NADIM研究中pCR与MPR值明显高于CheckMate-159研究中pCR与MPR值，提示免疫联合化疗新辅助治疗可能带来更好的疗效。NCT02716038为一项Ⅱ期临床研究，该研究入组了18例Ib期-Ⅲa期可手术的NSCLC患者，术前行4周期的atezolizumab联合白蛋白紫杉醇和卡铂新辅助治疗。其中14例患者的数据分析显示，术前8例（57%）患者影像学缓解，术后7例（50%）患者达到MPR，其中3例（21%）达到pCR，患者病理学缓解与PD-L1表达无关。NEOSTAR是一项Ⅱ期临床研究，对比了nivolumab单药新辅助与nivolumab+ipilimumab联合新辅助治疗Ⅰ期-Ⅲa期可手术NSCLC患者的疗效。研究入组36例患者，共32例患者完成新辅助治疗，7例（22%）达到了影像学缓解。在完成手术的26例患者中，5例（19%）患者达到了pCR，8例（31%）达到了MPR，总体耐受性良好，进一步分析显示免疫单药组与免疫联合组患者手术标本存活细胞比例的中位数分别为65%与27.5%，提示免疫联合组较单药组疗效更佳。目前还有很多类似研究正在进行（表 3），如评估durvalumab联合化疗新辅助+durvalumab辅助治疗NSCLC疗效的Ⅱ期临床研究NCT02572843，nivolumab联合CTLA-4双免疫治疗NSCLC的Ⅲ期临床试验NCT02998528等。

以上研究提示，免疫治疗早期使用更容易获得深度缓解甚至完全缓解。患者术前影像缓解率显著低于术后病理缓解率，表明影像学评估在判断新辅助免疫治疗疗效上仍存在不足。同时带给我们思考：免疫治疗如何与化疗药物或其他免疫治疗药物联合？用药次数和时机？新辅助免疫治疗后pCR的患者如何在术前得到明确及是否可避免手术治疗？在新辅助治疗中，驱动基因阳性患者能否突破免疫治疗疗效不佳的瓶颈？

## 5 结语

NSCLC免疫治疗的时代已经来临，以PD-1/PD-L1为靶点的免疫检查点抑制剂无论在晚期NSCLC二线治疗及一线治疗、局晚期患者辅助治疗还是早期患者新辅助治疗中都有获益，延长了患者生存，极大地改变了NSCLC治疗的格局^[28]^。但免疫治疗仍存在许多问题：①如何预测免疫治疗的疗效？目前PD-L1表达被认为是较为可靠的疗效预测生物标志物，TMB、微卫星高度不稳定性和肿瘤浸润淋巴细胞（CD8^+^ T淋巴细胞）也有一定的预测作用，但仍缺乏前瞻性研究证实。也有初步研究表明肠道微生物与免疫治疗疗效相关，通过调整肠道菌群来提高免疫治疗疗效也成为研究热点^[29]^。②传统的实体瘤疗效评价标准（Response Evaluation Criteria in Solid Tumor, RECIST）1.1不能准确地评价免疫治疗疗效，评价标准虽不断更新，如免疫相关缓解标准（immune-related RECIST, ir RECIST），免疫相关病理缓解（immune-related pathologic response criteria, irPRC），但仍存在局限性^[30, 31]^。③免疫治疗不良反应发生率较低，但有部分患者可能发生严重免疫相关不良反应（如心肌炎、肺炎、肝炎和肾炎等），甚至危及生命，如何预测、及时发现和治疗？④免疫治疗耐药涉及肿瘤细胞基因或信号通路表达异常、免疫逃逸、肿瘤微环境改变等多方面因素^[32, 33]^，如何处理免疫治疗耐药，也是目前免疫治疗的研究重点。⑤免疫治疗在驱动基因阳性患者的应用前景？
